# On the Use of Collodion in Ingrowing Nail

**Published:** 1851-10

**Authors:** M. Meynier


*On the Use of Collodion in Ingrowing Nail.* By M. Meynier, M. D.—M. Meynier treats this affection by pressing down the fleshy portion, and pouring in between this and the edge of the nail a small quantity of collodion, which soon solidifies, induces rapid cicatrisation of the ulceration, and, when the disease does not arise from an abnormal shape of the nail, procures a cure. M. H. Larrey has recently tried the plan in five cases, and succeeded in four of these.—*Bull. de Therap.*

